# Crosstalk Between the Tumor Microenvironment and Cancer Cells: A Promising Predictive Biomarker for Immune Checkpoint Inhibitors

**DOI:** 10.3389/fcell.2021.738373

**Published:** 2021-10-07

**Authors:** Xiaoying Li, Yueyao Yang, Qian Huang, Yu Deng, Fukun Guo, Gang Wang, Ming Liu

**Affiliations:** ^1^Department of Abdominal Oncology, West China Hospital, Sichuan University, Chengdu, China; ^2^National Engineering Research Center for Biomaterials, Sichuan University, Chengdu, China; ^3^School of Basic Medical Science, Chengdu University, Chengdu, China; ^4^Division of Experimental Hematology and Cancer Biology, Children’s Hospital Medical Center, Cincinnati, OH, United States

**Keywords:** tumor microenvironment, innate immunity, responsive or resistant biomarkers, immune checkpoint inhibitors, immune cells

## Abstract

Immune checkpoint inhibitors (ICIs) have changed the landscape of cancer treatment and are emerging as promising curative treatments in different type of cancers. However, only a small proportion of patients have benefited from ICIs and there is an urgent need to find robust biomarkers for individualized immunotherapy and to explore the causes of immunotherapy resistance. In this article, we review the roles of immune cells in the tumor microenvironment (TME) and discuss the effects of ICIs on these cell populations. We discuss the potential of the functional interaction between the TME and cancer cells as a predictive biomarker for ICIs. Furthermore, we outline the potential personalized strategies to improve the effectiveness of ICIs with precision.

## Introduction

**FIGURE 1:**
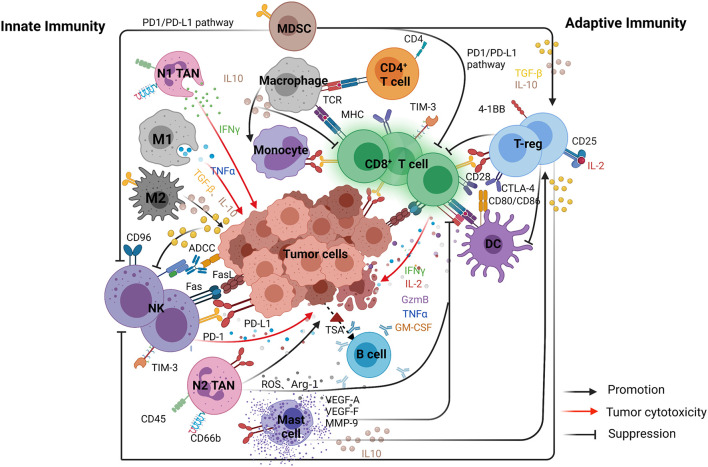
The main interactions of immune cells and the relationships between tumor and immune cells in the TME. The figure is divided into two parts to show the complementary and interdependent relationship between innate and adaptive immunity. Left, represents innate immunity included natural killer (NK) cells, dendritic cells (DCs), tumor-associated macrophages (TAMs), and tumor-associated neutrophils (TANs). Right, represents adaptive immunity included CD8 + cytotoxic T cells (CTLs), CD4 + T helper 1 (Th1), and B cells.

The tumor microenvironment (TME) is a key component of tumors that consists of various cell types including immune cells, endothelial cells, cancer-associated fibroblasts (CAFs) along with cytokines, chemokines and the extracellular matrix (ECM) (Hanahan and Weinberg, 2011). While certain cells in the TME have the potential to inhibit tumor development, other cells in the TME act synergistically with tumor cells to enhance tumor development (Figure 1). The tumor-promoting factors in the TME include immunosuppressive effector molecules and effector cells such as regulatory T cells (Tregs), myeloid-derived suppressor cells (MDSCs), and tumor-associated macrophages (TAMs) (Junttila and de Sauvage, 2013). The interaction between immunosuppressive TME and tumor cells regulates a range of cellular processes including tumor cell proliferation and metastasis. It also protects tumor cells from the clearance by immune effector cells. In addition, tumor cells can escape host immune reactions through immune checkpoints (Sharpe and Freeman, 2002; Francisco et al., 2010; Pardoll, 2012). Recently, immune checkpoint inhibitors (ICIs) targeting programmed cell death protein (PD-1), programmed death-ligand (PD-L1), and T-lymphocyte-associated protein 4 (CTLA-4) have shown efficacies in restoring antitumor immunity in multiple tumor types with tolerable adverse-event profiles (Zheng et al., 2017; Ren and Zhang, 2019; Yost et al., 2019). However, only a small proportion of patients showed strong responses to ICIs, as many patients developed primary or acquired resistance (Zheng et al., 2017). Thus, there is a need to find biomarkers to inform patient-specific treatments and to better understand the molecular mechanisms underlying the drug resistance. At present, predictive biomarkers are limited to PD-L1, tumor mutation burden (TMB) and MSI-H/dMMR. As these biomarkers are often unreliable, better biomarkers are highly desired. Given that the TME is a major obstacle to the success of cancer immunotherapy (Yost et al., 2019), one could imagine that the TME may serve as a predictive biomarker for ICIs. In this context, it is desired to better understand the complexities of immune cells within the TME, which may be achieved by using cutting-edge techniques such as single-cell RNA sequencing and mass cytometry (Spitzer and Nolan, 2016; Zheng et al., 2017; Simoni et al., 2018; Kiselev et al., 2019; Ren and Zhang, 2019; Zhang and Zhang, 2019). In this paper, we review the molecular heterogeneity of the TME and relate it to the unique challenges and opportunities for ICIs (Figure 1).

## The Biological Functions of Innate Immunity Within the TME in Cancer Immunotherapy

Anti-tumor immunity depends on tumor immunogenicity and the immune function of the host and other factors. The tissue of origin and occurrence of tumor cells leads to significant variations in immunogenicity and anti-tumor immune responses. Tumor immune responses include innate and adaptive response. Innate immunity develops gradually with age and involves the evolution and adaptation of the immune system. Generally, innate immune response is the first line anti-tumor effectors whilst the adaptive immune response plays more specific roles in the immune responses. However, innate immune response [dendritic cells (DCs), natural killer (NK) cells, TAMs, and tumor-associated neutrophils (TANs)] and adaptive immune response [CD8^+^ cytotoxic T cells (CTLs), CD4^+^ T helper 1 (Th1), and B cells] are complementary and interdependent (Tanaka et al., 1999).

### Dendritic Cells Within the TME Coordinate the Priming and Differentiation of T Cells

Dendritic cells are the central antigen-presenting cells (APCs) that can directly activate naïve T cells. DCs initiate the adaptive immune response and mediate interactions between innate and adaptive immune responses (von Andrian, 2002; Batista and Harwood, 2009). Following the activation of DCs, an inflammatory response is triggered and pro-inflammatory cytokines and chemokines are released to regulate immune function. The maturation and metastasis of DCs to the lymph nodes result in the activation of antigen-specific T cells that participate in adaptive immunity. DCs express high levels of adhesion molecules such as intracellular cell adhesion molecule 1 (ICAM-1) that allow strong binding to T cells and facilitate intercellular interactions (Segura et al., 2005). ICAM can participate in the innate immune response by recognizing and transporting antigens and can initiate an adaptive immune response, as well as enhancing antigen presentation and CTLs priming (von Andrian, 2002; Allan et al., 2006). Mature DCs express high levels of costimulatory molecules such as CD86, CD40, and CD80. CD40 and its ligand CD40L are also expressed on the surface of other APCs, such as B cells and macrophages, and act to significantly increase antigen presentation and co-stimulatory capacity (Schoenberger et al., 1998).

Dendritic cells present tumor-associated neoantigens through pattern recognition receptors (PRRs) in the early stages of tumorigenesis. Inter-tumoral stimulatory dendritic cells (SDCs) can stimulate CTLs and initiate immune responses against cancer. The activation of DCs is positively correlated with T-cell inflammatory status and response to PD-1/PD-L1 pathway inhibition (Barry et al., 2018).

A previous study investigated the TME in gastric cancer using single-cell RNA sequencing. The study reported that DCs infiltrating the TME (TIDCs) expressed chemokines such as CCL17, CCL19, CCL22, and IL-32 that helped recruit naïve T cells. These cells also displayed unique gene expression programs that differed from peripheral blood mononuclear cells (PBMCs) (Sathe et al., 2020). Although TIDCs have anti-tumor potential within the TME, the function of these cells is often impaired at the later stages of tumor development due to interactions among tumor cells and surrounding immune cells. In the early stage of tumorigenesis, PD-1 expression is low on TIDCs and cytokines such as IL-10, TGF-β, and arginase in the TME can upregulate the expression of immunosuppressive molecules, such as PD-1 and Tim-3, on TIDC cells. The overexpression of the molecules acts to convert DCs with anti-tumor potential into immunosuppressive DCs (Gardner et al., 2020).

Within the TME, cytokines produced by DCs may induce the activation and proliferation of Tregs (Figure 1). A novel subset of tolerogenic DCs can also promote the differentiation of T-regulatory cells (Tr1) through producing high levels of IL-10 (Gregori et al., 2010). DCs can secrete CCL22 that promotes interactions between DCs and Tregs *via* binding to its receptor CCR4. The recruitment of Tregs into the tumors cause immune suppression and downregulation of co-stimulatory molecules on DCs, causing CTLs dysfunction (Curiel et al., 2004; Bauer et al., 2014; Rapp et al., 2019). Ibrahim et al. (2012) showed that liver DCs with low lipid concentrations induced anergy in Tregs whilst DCs with high levels of lipids were immunogenic in many models and functioned to activate T and NK cells.

### Function of NK Cells in the TME

CD3^–^CD56^+^ NK cells can be divided into CD56^bright^ and CD56^dim^ subtypes. The main function of CD56^bright^ NK cells is to secrete cytokines whilst CD56^dim^ NK cells mainly mediate cytotoxicity. NK cells can kill targeted cells through several specific pathways including antibody-dependent cellular cytotoxicity (ADCC), the Fas-FasL pathway, the perforin-granzyme pathway and the secretion of pro-inflammatory cytokines, such as TNF, IFN-γ, GM-CSF, IL-6, and CCL5 (Voskoboinik et al., 2006; Guillerey et al., 2016; Habif et al., 2019).

In the early stages of tumor development, NK cells are the first line of defense against tumors. NK cells could migrate into tumors in response to chemokines secreted by DCs (Guillerey et al., 2016). The deletion or decreased expression of MHC molecules on the surface of tumor cells prevent the binding of NKs to the inhibitory receptor (killer inhibitory receptor, KIR) that inhibits the initiation of killing inhibitory signal. However, surface carbohydrate ligands can bind to the activated receptor (killer activation receptor, KAR) on the surface of NK cells to activate NK cells and exert a killing effect (Long, 2008; Thielens et al., 2012; Myers and Miller, 2021). Similar to TIDC, tumor-infiltrating NK cells have anti-tumor and anti-metastatic potential. In melanoma patients, it has been reported that NK cells positively regulated intratumoral SDCs through the production of cytokine FLT3L to enhance T cell responses (Veglia et al., 2018). In some tumors, tumor-infiltrating NK cells undergo phenotypic changes and dysfunctions compared to normal NK cells, which partially explains why NK cell-targeted therapies have low efficacy in some tumor types. The function of NK cells is suppressed by soluble regulatory factors (e.g., TGF-β) in the TME that can act directly on NK cells, leading to reduced cytotoxicity and cytokine secretion. Tumor-infiltrating NK cells’ function can also be dampened by NK cell-autonomous inhibitory checkpoints such as PD-1, TIGIT, CD96, TIM-3, LAG-3, CTLA-4, KIR2DL-1/2/3 and NKG2A (Guillerey et al., 2016). Previous studies have indicated that PD-1 expression on tumor-associated NK cells requires glucocorticoids (Quatrini et al., 2021; Figure 1). While ICIs may restore NK cell-mediated anti-tumor immunity (Kamran et al., 2017; Holmgaard et al., 2018; Sivori et al., 2020). Furthermore, anti-CD96 has been shown to stimulate NK cell function and improve the efficacy of ICIs (Du Four et al., 2016; Davis et al., 2017).

### Macrophages Within the TME Can Play a Tumor-Inhibitory or Tumor-Promoting Role

Tumor-associated macrophages are the main component of the TME where they can promote the formation of an immunosuppressive microenvironment or suppress tumorigenesis and metastasis, depending on the direct or indirect suppression of cytotoxic T-cell activity (Mantovani et al., 2017; Lin et al., 2019), accordingly divided into anti-tumor M1 and pro-tumor M2 types (Biswas and Mantovani, 2010; Figure 1). TAMs can suppress immune responses by producing immunosuppressive mediators/cytokines and also by expressing the inhibitory receptor, thus affect the infiltration of CTLs and suppress the function of CTLs by expressing the molecular triggers of checkpoint proteins (De Palma and Lewis, 2013; Ruffell and Coussens, 2015; Mantovani et al., 2017; Lin et al., 2019). As TAMs are the primary source of IL-10 in tumors and IL-10 can promote the expression of PD-L1 on monocytes, PD-L1^+^ monocytes can effectively inhibit tumor-specific T-cell immunity *via* the infiltration of Tregs and dysfunction of CD8 T-cells (Zhang et al., 2020; Figure 1). They can also promote tumor growth, indicating that PD-L1 expression on activated macrophages is a potential mechanism linking the pro-inflammatory response to the immune tolerance of the TME (Wynn et al., 2013; Romano et al., 2015; Zhang et al., 2020).

M1 macrophages, characterized with production of inflammatory cytokines and reactive oxygen/nitrogen species, showed anti-tumor effect and were valuable for host defense (Wynn et al., 2013). Moreover, it were reported to show high ratios in responder treated with ipilimumab (a fully human CTLA-4-specific mAb) in comparison with non-responder (Romano et al., 2015). The expression of PD-1 on TAMs and its interaction with PD-L1 on tumor cells may impair phagocytic capacity of macrophage. Interestingly, most of the PD-1 were found in M2 macrophages, and ICIs treatment could revert their function to M1 phenotype for killing tumor (Gordon et al., 2017; Figure 1). Tumor cells can cause macrophages to exhibit an immunosuppressive phenotype *via* releasing the autophagosome (TRAPs). The expression of PD-L1 and IL-10 can hinder the proliferation of CD4^+^ and CD8^+^ T cells, indicating that the TRAPs-PD-L1 axis is a promising option by simultaneously targeting autophagy and PD-L1 (Wen et al., 2018).

In contrast to M1 macrophages’ antitumor role, M2 macrophages predominate within the TME and can promote vascular growth, invasion and metastasis, and enhance chemoresistance (Xia et al., 2020). It has been reported that M2 polarization increased the expression of PD-L2 in TAMs that could lead to immune escape and tumor promotion through the PD-1 signal pathway (Huber et al., 2010). These data indicated that TAMs-targeting *via* blocking the CCL2-CCR2 axis was potential strategy to overcome immune evasion, and inhibiting the recruitment of TAMs might enhance the antitumor effect of CTLs in the TME (Yang et al., 2020). Besides, the ratio of M1/M2 macrophages can be used to evaluate the effectiveness of immunotherapy. Sathe et al. found that the TME could be reprogrammed based on the state of macrophages. The phenomena that TAMs differentiated from monocytes and retained basic features of macrophages were also found in normal tissues (Sathe et al., 2020).

The interaction of PD-L1 on T-cells with PD-1 on macrophage impacts the tolerance of macrophage differentiation (Diskin et al., 2020). Although macrophages have immuno-suppressive roles, the pro-inflammatory F480^+^MHCII^+^Ly6Cc low macrophage can induce interferon by secreting CXCL9. In patients treated with avelumab (an anti-PL-L1), the baseline levels of CXCL9 are related to clinical outcome, indicating that this subgroup of macrophages improves response rates to ICIs (Qu et al., 2020). Class IIa HDAC inhibition (TMP195) was used to modulate the phenotype of macrophages by Guerriero et al. (2017), they showed that the anti-tumor effect of TMP195 was enhanced when combined with T-cell checkpoint blockade. Macrophages may result in complete inability of T-cells to initiate an immune response against their target cells, therefore the effect of blocking immune checkpoints on monocytes within the TME may offer improved responses to ICIs.

### MDSCs Within the TME Are Associated With Resistance to Immunotherapy

Myeloid-derived suppressor cells originate from bone marrow progenitor cells that have not fully matured into granulocytes, monocytes and DCs (Weiskopf et al., 2016). MDSCs include groups of cells with different phenotypes that are biologically diverse in humans. In the TME, the proliferation of MDSCs is induced by various immune molecules produced by tumor, stromal and activated immune cells, such as GM-CSF and VEGF. In mice, MDSCs have been broadly identified as CD11b^+^ GR1^+^ cells, whilst in humans, they have been identified as LIN^–^ HLA^–^DR^–^ CD33^+^ cells (Veglia et al., 2018).

The primary function of MDSCs is to suppress CD8^+^ T-cell immunity by enhancing the expression of ROS, NO, arginase-1 and PGE-2 through PD-L1/PD-1 interaction (Veglia et al., 2018; Adeshakin et al., 2021; Figure 1). Other mechanisms include the induction of some immunosuppressive cells, the depletion of metabolites critical for T-cell function, the blocking of lymphocyte homing and the expression of ectoenzymes, etc. (Groth et al., 2019). MDSCs were recruited into the TME, their potent suppressive activities against effector lymphocytes may limit the efficacy of ICIs. The depletion of MDSCs is associated with the activation of CTLs’ responses. Several preclinical studies have shown that inhibition of MDSCs during immunotherapy could improve efficacy. MDSC-depleting chemotherapy increases the effects of anti-PD1 mAb whilst simultaneously improving CD8^+^ T-cell infiltration and effector cytokine secretion, thereby delaying tumor progression. Targeting MDSCs can improve patient response rate to immunotherapy. For example, tumor MDSCs can make TME immunosuppress through cell-specific mechanisms like TGF-β or nitric oxide in head and neck squamous cell carcinoma. And inhibiting CXCR1 and CXCR2 can eliminate MDSC accumulation and improve NK-Cell immunotherapy therapeutic efficacy (Greene et al., 2020). Apoptosis of MDSCs is caused by the high expression of TNF-related apoptosis-induced ligand receptors (TRAIL-Rs), and TRAIL-Rs’ expression is stronger at MDSCs in tumor sites. So that targeting TRAIL-Rs can lead to rapid and significant depletion of MDSCs, which can be used to improve the antitumor effect of various immunotherapy drugs (Condamine et al., 2014; von Karstedt et al., 2017). Thus, blocking the immunosuppressive environment mediated by MDSCs may be a potential area for the future development of effective treatments (Highfill et al., 2014; Du Four et al., 2016; Davis et al., 2017; Iida et al., 2017; Kamran et al., 2017; Veglia et al., 2018). Finally, high levels of circulating MDSCs in cancer patients often correlate with poor response rate to immunotherapy (Ai et al., 2018; Tavazoie et al., 2018), suggesting that MDSCs may serve as a predictive marker for ICIs.

### Mast Cells Have an Immunosuppressive Role in the TME

Mast cells are a group of innate immune sentinels. Mast cells secrete a variety of cytokines and participate in the regulation of key immune cell types including T, B, and APC cells (Voehringer, 2013). While mast cells may play an anti-tumor role in the TME, they are more appreciated to promote tumor progression. For example, mast cells have a pro-tumorigenic role in gastric cancer through the release of angiogenic (VEGF-A, CXCL8, and MMP-9) and lymphangiogenic factors (VEGF-C and VEGF-F) (Sammarco et al., 2019). Lv et al. found that mast cell infiltration into tumors through CXCL12-CXCR4-mediated chemotaxis resulted in immunosuppression (Lv et al., 2019). Mechanistically, mast cells secrete IL10, leading to increased numbers of Tregs in draining lymph nodes (Gan et al., 2012; Figure 1). Moreover, tumor-derived TNF-α activates NF-κB pathway in mast cells, causing mast cells to express PD-L1. In this context, inhibition of PD-L1 on mast cells may benefit cancer patients (Lv et al., 2019).

### Neutrophils Plays a Double-Edged Role in the TME

As a critical component of innate immunity, neutrophils are recruited to sites of inflammation by chemokines, cytokines and complement fragments (CXCL1, CXCL2, CXCL5, IL-8, C5a, and C3a) to enable host defenses against invading pathogens (Basu et al., 2002; Pagano et al., 2009; Ueha et al., 2011; Coffelt et al., 2016; Zhang and Houghton, 2021). On the other hand, the accumulation of peripheral blood polymorphonuclear neutrophils (PMN) within the TME promotes tumor growth and invasiveness in humans. TANs are CD45^+^CD66b^+^ (Zhang and Houghton, 2021) and can be classified as N1 and N2 subtypes. While N1 TANs exert anti-tumor activity through ADCC and proinflammatory factors production, such as IFN-γand MMP-8, in the innate immune response (Mihaila et al., 2021), N2 TANs promote tumor growth. Blocking TGF-β and inducing IFN-γ can cause N2 to convert to N1 (Fridlender et al., 2009).

N2 TANs are viewed as immunosuppressive cells (Kargl et al., 2019). In line with this, increased levels of neutrophils in tumors are associated with worse prognosis and poor outcomes in patients. This may be partially due to that PMN in the tumor matrix prevents T-cell infiltration. Concomitantly, increased neutrophil infiltration into tumors is associated with decreased efficacy of ICIs (Heng et al., 2009; Kargl et al., 2019; Schalper et al., 2020; Yuen et al., 2020). Vice versa, Kargl et al. (2019) found that higher ratio of CD8^+^ T cells to neutrophils was associated with more favorable responses. Mechanistically, tumor-derived GM-CSF induces PD-L1 expression in neutrophils through the Janus kinase (JAK) signal transduction and activator of transcription 3 (STAT3) signaling pathway (Figure 1). PD-L1^+^ neutrophils in turn inhibit T-cell immunity and promote tumor growth (Wang et al., 2017). These findings form a basis for the ongoing clinical trials (ClinicalTrials.gov NCT03161431, NCT03184870, NCT04123379) of a combination therapy by targeting neutrophil recruitment and ICIs.

## The Role of Adaptive Immune Response Cells Within the TME in Tumor Immunotherapy

In general, the adaptive immune response plays a more important role than innate immunity in a specific immune response. However, innate and adaptive immunity are complementary as the innate immune response acts to initiate the adaptive immune response. Tumor antigens can be classified as tumor-specific antigens (TSAs) and tumor-associated antigens (TAAs). TSAs are recognized by T cells and induce a cellular immune response whilst TAAs can be recognized by B cells and induce humoral immunity (Jhunjhunwala et al., 2021). It is believed that humoral immunity acts synergistically with cellular immunity to inhibit tumor growth with cellular immunity being the main force in anti-tumor immunity. CTLs and Th1 responses are the main mechanisms of cellular immunity (Vesely et al., 2011). On the other hand, tumor cells can evade the attack of the immune system through loss of tumor antigens, decreased expression of MHC class I molecules, downregulation of costimulatory signals, secretion of immune suppressants and induction of immunosuppressive cells such as Tregs. Enhancing and improving the adaptive immune response is a priority for the development of immunotherapies (Vesely et al., 2011).

### T-Cell Infiltration and Activation Within the TME Are Key Drivers of Anti-tumor Immune Response

CD8^+^ cytotoxic T cells are the main effector cells of anti-tumor immunity. The complete activation of T-cells depends on the activation of antigen and costimulatory signals as well as the action of cytokines. These processes form the basis of T-cell proliferation and differentiation. The first signal is the antigen stimulation signal that allows the initial activation of T-cells and upregulates the expression of activation-related molecules such as costimulatory molecules (Tanaka et al., 1999). T-cells and APCs have multiple pairs of costimulatory molecules expressed on their surfaces. Interactions between costimulatory molecules, such as CD80 (B7-1), CD86 (B7-2), and CD28, are essential for the specific activation of T-cells to promote IL-2 transcription and stabilize mRNA (Watts and DeBenedette, 1999). Other costimulatory molecules are 4-1BB and 4-1BBL, ICOS and ICOSL, CD40 and CD40L. Fully activated T-cells express co-inhibitory receptors such as PD-1 and Tim-3. The balance between positive costimulatory and negative costimulatory molecules affects the activation of T cells. ICIs can increase the ratio of costimulatory to co-inhibitory mediators (Im et al., 2016). Inhibitory checkpoints like PD-1 and CTLA-4 have been targeted to relieve the depletion of CD8^+^ T-cells and have shown efficacy in the clinic (Croft, 2003; Farhood et al., 2019; Kallies et al., 2020; Figure 1). Other checkpoint receptor targets such as TIM-3, VISTA, LAG-3, TIGIT, and CD96 are currently being explored for clinical applications (Anderson et al., 2016; Dougall et al., 2017; Kakavand et al., 2017; Qin et al., 2019; Tu et al., 2020).

CD8^+^ cytotoxic T cells will enter an exhausted state as antigens and inflammation persist in the TME leading to T-cell dysfunction (Zheng et al., 2017; Bengsch et al., 2018; Sathe et al., 2020). By blocking the PD-1 inhibitory pathway, exhausted CD8 T (Tex) characterized by loss of the effector functions can be reinvigorated, indicating the therapeutic potential of improving immune control (Im et al., 2016; McLane et al., 2019). The most critical aspect of anti-PD-1 therapy is the survival of effector T-cells that are active in the TME (Beltra et al., 2020). In a study conducted in metastatic melanoma patients, patients who responded to pembrolizumab (anti-PD-1 therapy) showed proliferation of inter-tumoral CD8^+^ T-cells that directly correlated with tumor regression (Tumeh et al., 2014). According to other reports, sufficient T-cell infiltration is also a prerequisite for tumor responses to PD-L1 blockade. These data indicate that targeting LIGHT might increase responses to checkpoint blockades by creating a T-cell inflamed microenvironment that can also overcome tumor resistance to checkpoint blockade in non-T-cell inflamed tumors (Tang et al., 2016). Furthermore, pre-existed tumor-specific T-cells may have limited reactivation ability, whilst T-cell clones that have just entered the tumor may account for the response of T-cells to checkpoint blockade (Yost et al., 2019).

The relationship between CD8^+^ effector T-cells and PD-1 expression on Tregs in the TME could be used to predict the efficacy of anti-PD-1 immunotherapy. PD-1^+^ Tregs in tumor-infiltrating lymphocytes (TILs) can be used as therapeutic targets to enhance the clinical efficacy of ICIs. In addition, PD-1 expression by Tregs in TILs may explain the resistance to PD-1 blockade therapies (Kumagai et al., 2020; Figure 1).

An in-depth analysis of PD-1-CD8^+^ TIL found that these three subgroups shared common characteristics with naive, memory and effector CD8^+^ T-cells. Also, the proportions of these cell types may change the response to different ICIs in different cancers. Increases in the number of memory precursor-like CD8^+^ T-cells after treatment are related to a good prognosis and response to ICIs. Also, the transcription factor Tcf7/Tcf1 is a key regulator of this subgroup. If it is not expressed, checkpoint blockade and innate agonist immunotherapy can fail (Kurtulus et al., 2019).

Programmed death-ligand can be detected both on tumor cells and in the immune stroma. Higher CD8^+^ T-cell densities are accompanied by higher PD-L1 expression, indicating a possible mechanism of adaptive immune resistance (Thompson et al., 2017). TGF-β1 derived from tumor cells promotes the Smad3-dependent expression of PD-1 and Smad2-dependent dysfunction of CTLs, whilst PD-1 blockade cannot reverse this immunosuppressive environment (Shen et al., 2020). Diskin et al. (2020) found that PD-L1^+^ T-cells suppressed neighboring T-cells in the TME. The interaction between PD-L1 and PD-1 induces inhibitory signaling in T-cells and drives TH17 differentiation and signaling pathways related to T-cell immunogenicity such as STAT1, AKT, p38, and ERK. PD-L1^+^ T-cell expression has multiple effects on the innate and adaptive immune tolerance, immune synaptic cell crosstalk and TME signal transduction in cancer patients. These interactions may play important roles in immunotherapy response and drug resistance in cancer patients.

### Th Cells Are Indirectly Involved in Anti-tumor Immune Effects in Tumor Immunity

CD4^+^ Th cells include T helper type 1 (Th1), Th2, and Th17 cells (Ruterbusch et al., 2020). Although CD4^+^ Th cells are not the main effector cells of cellular immunity, CD4^+^ Th cells assist in activating CTLs and producing cytokines and chemokines that are indirectly involved in anti-tumor immune effects (Borst et al., 2018). For example, Th1 can influence APC antigen processing and also secrete chemokines including IL-2 and IFN-γ to recruit CTLs and NK to exert a local anti-tumor effect and to stimulate DC cells (Knutson and Disis, 2005). Cytokines secreted by Th2 cells are important for DCs maturation, clonal proliferation and class switching of B-cells, therefore these changes also promote humoral immunity (Ruterbusch et al., 2020). Th17 was initially identified as a CD4^+^ T-cell that secretes IL17 which is a separate lineage to Th1 and Th2 cells. It was found that Th17 mainly secretes IL-17A, IL-17F, and IL-22 which recruit and activate neutrophils. Th17 may also promote angiogenesis and participate in tumor formation, yet it remains unclear whether Th17 is predominantly tumor-suppressive or tumor-promoting (Weaver et al., 2007; Silva-Santos, 2010).

### Regulatory T Cells Within the TME Limit the Efficacy of ICIs

T-cells that constitutively express CD4 and CD25 are essential for maintaining self-tolerance and are therefore termed regulatory T cells (Tregs). The function of Tregs is defined by the transcription factor Foxp3 (Samstein et al., 2012; Bin Dhuban et al., 2014). By single-cell sequencing, it was showed that Tregs were significantly enriched in the TME in gastric cancer compared to normal tissue and contributed to an immunosuppressive TME. Also, Tregs express several immune checkpoints such as CTLA-4 and costimulatory molecules such as 4-1BB that are potential targets for regulating their functions (Sathe et al., 2020; Figure 1). The number of Tregs expressing immunosuppressive receptors in tumors is correlated with the activation and proliferation of CD4^+^ and CD8^+^ effector T-cells. Along with increases in Tregs, the cytokines (such as IL10 and TGF-β) that inhibit the effects in tumors are also up-regulated.

In a preclinical model, anti-CTLA-4 mAb has been shown to effectively induced the depletion of Tregs *via* an Fc-dependent mechanism in the TME but not in the peripheral lymphoid organs (Tang et al., 2018). This may be because the expression of CTLA-4 by Tregs in the tumor may be significantly higher than in the peripheral lymphatic organs. In human tumors, anti-CTLA-4 immunotherapy increases infiltration of inter-tumoral CD8^+^ and CD4^+^ cells without depleting FOXP3^+^ cells (Sharma et al., 2019).

The blocking of PD-1 and CTLA-4 can increase the ratio of effector T-cells to Tregs in tumors. However, the blocking of PD-1 is not entirely positive for T-cells. ICIs can also activate and stable Tregs. Comparison of GC tissue samples before and after anti-PD-1 mAb therapy found that the infiltration of Tregs was associated with rapid disease progression known as hyper progressive disease (HPD). Moreover, PD-1 blockade by enhancing the proliferation and immunosuppressive activity of PD-1^+^ Tregs in humans and mice inhibits antitumor immunity and enhances the suppressive activity of Tregs. The presence of actively proliferating PD-1^+^ Tregs in tumors may be a reliable biomarker for HPD and can be used to guide the use of PD-1 blockade (Kamada et al., 2019). When the number of effector cells increases, their activity also increases and Tregs are eliminated to maximize the antitumor effect.

CD25 expression is largely restricted to tumor infiltrating Tregs in mice and humans. Anti-CD25 antibody enhances binding to activate Fc gamma receptor (FcγRs), depleting tumor-infiltrating Tregs and increasing effector cells to Tregs ratios. The changes synergize with anti-PD-1 to eradicate established tumors (Arce Vargas et al., 2017). Eliminating Tregs in the TME could be an effective cancer treatment and prevent HPD during anti-PD-1 therapy. Fc-mediated depletion of inter-tumoral regulatory T-cells may be effective in combination with immunotherapy.

### B-Cells in the TME Play Controversial Roles in Tumor Immunity

The role of T-cells in tumor immune monitoring is well known, however, the role of B-cells in the TME has not been extensively studied. B-cells mediate humoral immunity mainly through the production of antibodies and exert immune-regulatory functions by producing cytokines. The role of B-cells in tumor immunity is multifaceted. Antibodies can mediate ADCC and cytokines (such as IL6, IL10) are involved in regulating the function of macrophages and dendritic, NK and T-cells (Fridman et al., 2021). For example, Bregs can secrete inhibitory cytokines, such as IL-10, TGF-β, and IL-35, that inhibit the physiological functions of effector CD4^+^ T cells by direct or indirect means. They can kill macrophages, dendritic cells and other immune cells during tumor development (Dasgupta et al., 2020). However, in breast cancer, B-cells express activated markers and produce cytokines and immunoglobulins to activate the humoral immune responses to effective anti-tumor immunity (Garaud et al., 2019).

B-cells may also play a prominent role in tumor infiltration and negatively regulate tumor growth. Higher tumor-infiltrating B-cells in HPV-associated oropharyngeal squamous cell carcinoma were associated with high CXCL9 production and high levels of tumor-infiltrating CD8 T-cells. These data indicated CD8 T-cells might be recruited *via* CXCL9 (Inoue et al., 2006; Hladíková et al., 2019). In addition, B-cells play roles in the formation of tumor-associated tertiary lymphoid structures (TLS) that may promote the induction of T-cell phenotypes required for response to ICIs. However, specific B-cell subsets are associated with immune-related adverse events (irAEs) in ICIs treatments (Willsmore et al., 2020). Recently, by bulk RNA sequencing, it was shown that B-cells were different in the tumors of responders versus non-responders during ICIs treatment, implying that B-cells were predictive and potential therapeutic targets (Helmink et al., 2020).

## The Complexity of Immune Effector Molecules With the TME

Immune molecules produced by immune cells and enzymes are involved in the anti-tumor effects of the immune response. Tumor cells can activate B-cells to secrete antibodies with an anti-tumor effect because of the expression of tumor antigens. These antibodies can exert their anti-tumor effect. In some cases, tumor-specific antibodies interfere with the specific killing effect of tumor cells. This growth-promoting antibody is called the enhancing antibody. Also, antibodies can change or lose the adhesion characteristics of tumor cells to promote tumor cell metastasis (Vesely et al., 2011). Other immune effector molecules in anti-tumor immunity, such as IFN and TNF, complement molecules and various enzymes have non-specific inhibitory or killing effects on tumor cells (Demaria et al., 2019). Prolonged exposure of tumor cells to a microenvironment in which IFN-γ is presented induces high expression of PD-L1 and IDO1. These tumor cells, in turn, inhibit the release of IFN-γ by effector T-cells, leading to T-cell depletion and tumor progression.

Chemokines are essential for immune cell recruitment and the therapeutic efficacy of ICIs. For example, CXCR3 and its ligand CXCL9 were critical for a productive CD8^+^ T cell response in tumor-bearing mice treated with anti-PD-1, indicating that the CXCR3 chemokine system was an indicator of the clinical sensitivity to anti-PD-1 mAb. Mechanistically, inter-tumoral CD103^+^ dendritic cells produce CXCL9, facilitating interactions between DCs and T-cells within the TME (Chow et al., 2019). Moreover, after dual PD-1/CTLA-4 blockade, the CXCR3 ligands, CXCL9, and CXCL10 were significantly up-regulated, indicating that macrophage-derived CXCR3 ligands were essential for the efficacy ICIs (House et al., 2020).

Interleukins are the most common and most diverse cytokines in the TME. Different interleukins have completely different effects on tumors, but the same interleukin can also have double-sided effects on tumors. For example, IL22 has been found to induce endothelial cell proliferation and promote the formation of blood vessels in tumors (Protopsaltis et al., 2019). While IL2 is a cytokine that has a positive role in immune activation by activating NK cells and CTLs to cause tumor regression. However, IL2 also can bind to the IL2Rα receptor on Tregs to stabilize and expand Tregs and play a negative role (Lim et al., 2020).

Intra-tumor expression or inhibition of cytokines or chemokines is a promising approach for tumor therapy. IL-12 is a cytokine that activates both innate and adaptive immunity, partially due to IFN-γ secretion from NK cells, CD8^+^ and CD4^+^ T cells. Although in a past clinical study, systemic administration of IL-12 caused severe adverse events, IL-12 remains an attractive candidate for cancer immunotherapy. Vaccinia virus encoding both IL-7 and IL-12 completely changed the tumor immune microenvironment by boosting the inflammatory immune status, which showed beneficial systemic antitumor efficacy and markedly improved the sensitivity of solid tumors to systemic anti-PD-1 and anti-CTLA4 (Nakao et al., 2020).

## The Complex Interactions Among Stroma and Immune Cells as Well as Tumor Cells in TME

**FIGURE 2:**
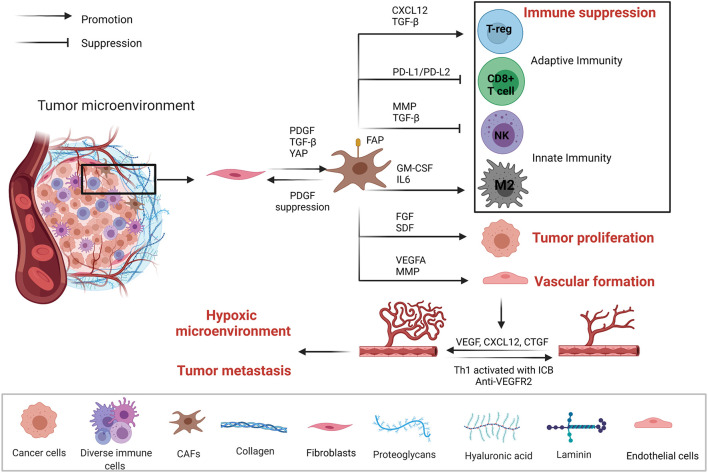
Mechanisms of tumor-associated fibroblasts (CAFs) and vascular endothelial cells affecting the TME. CAFs, vascular endothelial cells, tumor cells, and immune cells are in a dynamic relationship. CAFs through the secretion of cytokines and PD1/PDL1, PDL-2 pathways form the immunosuppression microenvironment to promote tumor proliferation. Tumor microenvironment regulates vascular endothelial cell proliferation and the formation of blood vessels through the secretion of growth factors and cytokines. Eventually, TME becomes hypoxic, accelerating tumor metastasis.

The development of solid tumors is accompanied by excessive deposition of ECM, abnormal tissue pattern and activation and enrichment of CAFs. A large amount of evidence has shown that the key components of stroma in the TME not only were conducive to the growth and metastasis of tumor cells but also hindered immune cell infiltration and affected the anti-tumor immune response (Figure 2). Cancer-associated fibroblasts are highly heterogeneous for their dynamic origins, by signals like TGF-β, PDGF, and YAP in tumors inducing fibroblasts into activation state (Kalluri, 2016; Biffi and Tuveson, 2021). CAF is closely related to the changing state of ECM. On the other hand, ECM affects the activation of CAFs and their functional exertion. Both CAFs and ECM play important pro-tumorigenic and antitumorigenic roles in the creation of TME, especially in solid tumors (Cox, 2021). CAFs and ECM dynamically interact with the tumor cell, which is not only important pathological features of solid tumors but also important driving forces for malignant tumor development (Najafi et al., 2019; Yoshida, 2020), such as changing the microenvironment, regulating paracrine signals through inflammatory cytokines, controlling tumor immune responses, depositing different extracellular matrix components, stimulating angiogenesis, providing scaffolds for tumor metastasis and invasion and regulating malignant cell metabolism (Levental et al., 2009; Erdogan and Webb, 2017; LeBleu and Kalluri, 2018; Demircioglu et al., 2020). With the secretion of cytokines like CXCL12 and IL-6, CAFs regulate the recruitment of macrophages and their contribution to tumor-promoting M2 type differentiation, thus affecting innate immunity (Ruffell and Coussens, 2015). Moreover, CAFs and ECM allow the TME to be maintained in a state of immunosuppression, thus greatly limiting the effect of cancer immunotherapy. For instance, FAP-positive CAFs suppress the anti-tumor efficacy by expressing CXCL12, which causes T-cells in tumors exclusion and regulates adaptive immunity. The removal of CAFs or CXCR4 antagonists causes tumors to internal T-cell immersion and enhanced PD-L1 antibody immunotherapy (Feig et al., 2013). As TGF-β, PDGF, and FGF2 are the main activating factors of CAFs, strategies targeting CAFs and ECM remodeling like re-educating of the tumor stroma have also made some progresses. For example, suppressing PDGF signal pathway can make CAFs reversed to normal tissue fibroblasts and inhibit tumor growth, thus better regulating therapeutic efficacy and sensitivity (Pietras et al., 2008; Kalluri, 2016). The normalization of CAFs and ECM is a promising direction in tumor therapy and potential stromal targeting cancer therapies are underway.

Vascular endothelial cells are a major component of non-immune stromal cells. However, during tumor angiogenesis, vascular endothelial cells do not form a dense structure, but form a loosely structured, highly permeable vessel, thus affecting the infiltration of lymphocytes. Moreover, the permeability of blood vessels is closely related to the hypoxic microenvironment inside the tumor, tumor metastasis and tumor response to drugs (Figure 2). By normalizing vasculature may mitigate hypoxia and facilitate infiltration of lymphocytes. For instance, Th1 activated with ICB plays a pivotal role in tumor vessel normalization, and ICIs-activated CD4^+^ T lymphocytes increases vessel normalization. Moreover, subgroup TH1 cells that secrete interferon-γ play more critical roles in vessel normalization (Tian et al., 2017). CAFs can induce angiogenesis by secreting cytokines such as VEGF, CXCL12 and CTGF (Wu et al., 2021). VEGF/VEGFR-2 signaling induces the proliferation, migration, and angiogenesis of vascular endothelial cells, but also elevates the permeability of blood vessels. Anti-angiogenic treatment by blocking VEGF has shown anti-tumor effect by disturbing angiogenesis (Dvorak, 2002). In HCC murine models, dual PD-1/VEGFR-2 antibodies overcome anti-PD-1 treatment resistance *via* promoting CD4^+^ cell-mediated vessel normalization and reducing negative regulatory components like Tregs and CCR2^+^ monocytes, thus converting TAMs from M2 to M1 type,as well as facilitating infiltration and activation of CTL. Therefore, synergistic ICIs with anti-angiogenesis may improve sensitization of the tumors to ICIs (Shigeta et al., 2020; Figure 2).

## Targeting TME in Combination With Immunotherapy

Cancer immunotherapy using ICIs and CAR-T cells has developed rapidly, and has revolutionized cancer therapy. Immunotherapies targeting TME are also emerging, for example, targeting CTLs by blockading inhibitory checkpoints or by activating stimulatory checkpoints. Since the approval of Ipilimumab by the FDA in 2011, anti-CTLA-4 and anti-PD-1/L1 have demonstrated efficacy in various tumor types (Hodi et al., 2010). Different cells within the TME have roles in promoting or inhibiting tumor growth. CTLA-4 can be expressed on the surface of CTLs, NK cells and Tregs. CTLA-4 monoclonal antibody can relieve the inhibitory effect of CTLA-4 on CTLs and NK cells (Krummel and Allison, 1995; Benson et al., 2010). Other emerging ICIs, such as anti-tim3, can also play critical roles in driving anti-tumor immune responses (Gleason et al., 2012; Anderson, 2014). These drugs are able to indirectly improve the anti-tumor activity of CTLs and NK cells by reducing the cell number of Tregs (Bulliard et al., 2013). Specific targeting on different cells with ICIs may have synergistic effects. Although only drugs that activate T-cells have been brought to market, the scope for other combinations will be rapidly developed in clinical trials to explore the impact of these drugs within the TME (Table 1).

**TABLE 1:** Landmark and ongoing trials of targeting tumor immune microenvironment synergize ICIs.

**Treatment type**	**Treatment mechanism**	**Trial name (NCT number)**	**Current status**
CTLs-based therapy with ICIs	Blockade of inhibitory checkpoints: Anti-PD-1/Anti-PD-L1/Anti-CTLA-4/Other ICIs, enabling tumor-reactive T cells to overcome regulatory inhibitory mechanisms.	NCT02453594/NCT00094653/NCT01927419 NCT01721746/NCT02108652/NCT02125461	Combination therapies to overcome tumor immune evasion, other ICIs have emerged as potential targets.
NK cells -based therapy with ICIs	1. Targeting NK inhibitory molecules 2. Targeting NK cell activating signals 3. Adoptive NK cells therapy	NCT02665650/NCT03586869/NCT04261439 NCT03387085/NCT04143711/NCT03841110	Natural killer (NK) cell-based therapies are emerging as safe and effective treatments for some cancers, auxiliary methods for enhancing the therapeutic activity of NK cells include immune- checkpoint inhibitors
TAMs-targeted therapy with ICIs	1. Anti-CSF-1 antibodies and CSF-1R inhibition to deplete macrophages 2. Agonistic anti-CD40 or inhibitory anti-CD47 antibodies to stimulate macrophages 3. Modulation of macrophage phenotype 4. Eliminating TAMs already present in the TME 5. Inhibition of monocyte recruitment 6. Reprogramming of TAMs	NCT04123379/NCT03767582/NCT03059147 NCT02826486/NCT02907099/NCT04058145 NCT02777710/NCT02323191/NCT03768531 NCT02554812/NCT03558139/NCT03869190 NCT02807844/NCT02890368/NCT02663518 NCT01103635/NCT03123783/NCT02304393 NCT04116320/NCT03435640/NCT04193293	To convert its immunosuppressive ability to its potential immunostimulatory function, which is beneficial to the current ICI-based immunotherapy
MDSC-targeted therapy with ICIs	1. Decrease MDSCs recruitment 2. Promote MDSC depletion 3. Reprogram MDSCs to enhance anti-tumor immunity	NCT03214666/NCT02403778	Combined treatment with ICIs along with small molecule inhibitors to precise target MDSC remains challenge.
Neutrophils-targeted therapy with ICIs	Inhibition of various chemokines(IL8, Arg1, CXCR2, IL1β)to retard PMN recruitment and function.	NCT03161431/NCT03184870/NCT03473925 /NCT04123379/NCT02903914/NCT03631199	Pre-clinical studies by targeting neutrophil recruitment and neutrophil immunosuppressive function are currently under to complement the ICIs monotherapy
Tregs-targeted therapy with ICIs	Depletion of Tregs synergizes with ICIs to eradicate established tumors, for example blocking CCR4 and Tregs chemotaxis; blocking various chemokines and chemokine receptor (TGF-β, IL-10 and IL-35)	NCT02476123/NCT02705105/NCT02444793/NCT02301130/NCT02503774	Through depleting Tregs depletion combination with ICIs, eliminating Tregs mediated resistance
Stroma–targeted therapy/anti-angiogenesis with ICIs	Stroma–targeted therapy by reversing CAFs and ECM to antitumorigenic roles. By normalizing vessel formation, reducing negative regulatory components like Tregs, promoting of CTL infiltration and activation	NCT02681549/NCT02337491/NCT02348008 NCT03475004/NCT02443324/NCT03650764 NCT02210117/NCT02873962/NCT03452579 NCT02999295/NCT03502746/NCT02336165	Potential stromal targeting cancer therapies are underway. ICIs coupling with anti-angiogenesis have already shown efficacy in the clinic, but deeper understanding of the immunomodulatory capacity still unsatisfactory

Not all patients are responsive to ICIs and primary resistance may be due to low levels of lymphocytes within the TME (Ochoa de Olza et al., 2020). Additionally, patients who respond to ICIs also have the possibility to ultimately develop acquired resistance. This occurs through several mechanisms such as downregulation of the antigen presentation machinery, loss of IFN-γ sensitivity, neoantigen depletion, tumor-mediated immunosuppression, and the expression of other inhibitory checkpoints (Schoenfeld and Hellmann, 2020). Although the mechanisms of immune checkpoints are largely dependent on CD8^+^ effector cells, an increasing number of studies have found that the response to ICIs is correlated with other components of the TME. The combination of ICIs with agents that target the TME components has major potential to optimize therapeutic efficacy and overcome challenges associated with drug resistance and tumor recurrence. TGF-β promote immune evasion in TME, thereby limiting the efficacy of ICIs. And it has been found that TGF-β inhibitor combined with PD-L1 antibody inhibits tumor metastasis of colorectal cancer in preclinical mouse models (Tauriello et al., 2018).

## Discussion

So far, predictive biomarkers, such as PD-L1, TMB, and microsatellite instability (MSI) et al., are often not reliable, and better sensitive biomarkers are highly desirable. Higher PD-L1 expression on tumor cells is likely to increase susceptibility to ICIs and achieve an objective response (Topalian et al., 2012). In addition to PD-L1 expression on the cell surface, metastatic melanoma with high level of exosomal PD-L1 (a circulating form of extracellular PD-L1) are positively responsive to ICIs therapy. Consumption of PD-L1 inhibitors by soluble PD-L1 may contribute to further understanding the mechanisms of tumor resistance to PD-L1 inhibitors. PD-L1 in tumor-derived exosomes can assist tumor cells in immune escape, therefore the combinations of small molecule drugs that inhibit the release of exosomes with ICIs may be used to improve therapeutic efficacy. It has been indicated that exosomal PD-L1 might be more predictive and facilitate the identification of responders and non-responders (Chen G. et al., 2018; Poggio et al., 2019; Orme et al., 2020). Tumor mutation burden (TMB-H) was supposed to be a predictive biomarker for the efficacy of response to ICIs in multiple cancer types (Samstein et al., 2019; Jardim et al., 2020), but a recent study indicated that TMB-H tumors indeed have higher objective response rates (ORRs) in melanoma, lung and bladder cancers, but failed to show the same predictive efficacy among breast cancer, prostate cancer and gliom. Moreover, the predictive power of TMB in dual anti-PD-1/CTLA-4 checkpoint blockade is less satisfactory than monotherapy (Klein et al., 2021; McGrail et al., 2021). Besides, the cutoffs of TMB-H are not universal. Generally, TMB of 10 or more mutations per megabase is more likely to have higher response rates after ICIs treatment (Valero et al., 2021). Other specific mutations may also provide insights into the effects of immunotherapy, such as MMR, PRKDC, HED and POLE (Le et al., 2015; Chowell et al., 2019).

Tumor cells are inextricably linked to their microenvironment from occurrence, development, growth, metastasis and invasion. They can be further divided into “hot tumor” and “cold tumor” according to the types of invading immune cells, as the suppressive immune microenvironment of tumors limits the infiltration of effector immune cells. Inhibitory changes and the heterogeneity in the TME are important factors that can promote tumor progression and affect responses to immunotherapy. TILs can exert an antitumor effect through the host cellular immune response. It was reported that TIL levels could predict tumor control in EBV-positive gastric cancers (Kang et al., 2016). PD-L1 positivity has been shown to correlate with the presence of high TIL infiltration, as a higher TIL density was also associated with a lower risk of progression in gastric cancer patients (Dai et al., 2016). The infiltration of multiple immune cell types, such as TAMs and Tregs, may explain the limited efficacy of ICIs based on subgroup analysis of immune cell infiltration. The TME is a complex integrated system. Genomic and transcriptomic analysis offer a multifaceted view on TME and provide approaches for precision medicine (Cieślik and Chinnaiyan, 2018; Beaubier et al., 2019; Rodon et al., 2019). For example, by transcriptomic analysis, over 10,000 cancer patients were classified into four distinct TME subtypes. Among them, the immune-favorable TME subtypes were more likely to be susceptible to ICIs than the suppressive subtypes. So it has been demonstrated that visual tools containing transcriptomic and genomic data could help us better understand the tumor framework, mutational load, immune composition, anti-tumor immunity and immunosuppressive escape mechanisms (Bagaev et al., 2021). Comprehensive analysis and visualization may also help us identify biomarkers and guide therapeutic decision.

Interventions have been applied to target different components of the TME, aiming to convert a tumor-promoting into a tumor-suppressive TME. That is also optimal for ICIs-based therapies. Strategies targeting the TME were used to overcome the tumor resistance to immunotherapy, for example, through CD40-mediated immune cell activation (Diggs et al., 2020), tumor-penetrating peptide iRGD-mediated tumor-specific lymphocyte infiltration (Ding et al., 2019), combined inhibition of CD38 and PD-L1 (Chen L. et al., 2018) or radiotherapy combined with immunotherapy (Yu et al., 2021). TGF-β released from cancer cells, stromal fibroblasts and other cells can shape the architecture of the TME by suppressing the antitumor activities of immune cells to attenuate the anti-tumor efficacy of ICIs (Mariathasan et al., 2018; Derynck et al., 2020). The blockade of TGF-β signaling may alter the immune microenvironment, making it more amenable to immunotherapy and offering synergy with ICIs. These changes may augment intra-tumoral CD8 T-cell proliferation, reduce exhaustion and evoke pro-inflammatory cytokines that can promote antitumor immunity (Greco et al., 2020).

In this review, we focus on the TME and its interactions with ICIs. However, the immunosuppressive TME is a complex network regulated by a variety of immunosuppressive signals that are dynamic and continuously changing. Targeting single specific immunosuppressive signal may not be optimal to achieve long-term efficacy. Immunotherapy biomarkers are supposed to associate with advantages and shortcomings, such as positive biomarkers to assess the benefit of treatment while negative biomarkers predict the patient’s immune resistance, hyper progression, severe toxicity, *etc*. As a combination of TMB, PD-L1 and neutrophil-lymphocyte, ratio (NLR)status has shown improved predictive power (Bruni et al., 2020; Kao et al., 2021). Screening of sensitive biomarkers like exosomal PD-L1, TMB, specific mutations and combined assessment of multiple biomarkers may be the future research directions. Designing multiple combined immunotherapy strategies and exploring new immunotherapy targets are the potential priority areas in scientific researches and clinical trials.

## Author Contributions

ML put forward the conception and design. GW completed the critical comments and revision. XL drafted the manuscript. YY, QH, YD, and FG collected, analyzed the data, and prepared the table. All authors contributed to the article and approved the submitted version.

## Conflict of Interest

The authors declare that the research was conducted in the absence of any commercial or financial relationships that could be construed as a potential conflict of interest.

## Publisher’s Note

All claims expressed in this article are solely those of the authors and do not necessarily represent those of their affiliated organizations, or those of the publisher, the editors and the reviewers. Any product that may be evaluated in this article, or claim that may be made by its manufacturer, is not guaranteed or endorsed by the publisher.
